# An Overview of Models for Response Times and Processes in Cognitive Tests

**DOI:** 10.3389/fpsyg.2019.00102

**Published:** 2019-02-06

**Authors:** Paul De Boeck, Minjeong Jeon

**Affiliations:** ^1^Department of Psychology, Ohio State University, Columbus, OH, United States; ^2^KU Leuven, Leuven, Belgium; ^3^Graduate School of Education and Information Studies, University of California, Los Angeles, Los Angeles, CA, United States

**Keywords:** response time, response accuracy, cognitive tests, cognitive processes, psychometric models, local dependencies, automated and controlled processes

## Abstract

Response times (RTs) are a natural kind of data to investigate cognitive processes underlying cognitive test performance. We give an overview of modeling approaches and of findings obtained with these approaches. Four types of models are discussed: response time models (RT as the sole dependent variable), joint models (RT together with other variables as dependent variable), local dependency models (with remaining dependencies between RT and accuracy), and response time as covariate models (RT as independent variable). The evidence from these approaches is often not very informative about the specific kind of processes (other than problem solving, information accumulation, and rapid guessing), but the findings do suggest dual processing: automated processing (e.g., knowledge retrieval) vs. controlled processing (e.g., sequential reasoning steps), and alternative explanations for the same results exist. While it seems well-possible to differentiate rapid guessing from normal problem solving (which can be based on automated or controlled processing), further decompositions of response times are rarely made, although possible based on some of model approaches.

## Introduction

Cognitive tests are meant to measure abilities. Abilities refer to levels of performance, whereas processes are the activities involved in reaching a performance outcome. Typically, cognitive tests do not yield process measures. It is perfectly possible to measure an ability without knowledge of the processes that are involved, but then the resulting measure only describes the level of performance, which is not always satisfying because it leaves why questions unanswered. Explanation requires a narrative of how something comes about. Processes provide such a narrative. Processes do not only help for understanding, they also help for more informative feedback and knowing the processes may help for interventions and remediation. Process information is also relevant to make validity inferences in the positive sense if the inferred processes support the interpretation of the intended ability, and in the negative sense, for example, because unintended processes can invalidate a measurement result. An important example of an invalidating process is guessing. Like it is possible to measure without investigating processes, it is also possible to investigate processes without measuring the related abilities, and a combination of the two is also possible.

Processes have the intrinsic feature that they take time. Therefore, response times are natural and evident kinds of data to investigate processes. Other kinds of data can also be informative regarding processes involved in reaching or not reaching a certain performance level. In fact, the responses themselves may be informative. For example, based on a cognitive theory stipulating the processes involved in finding the correct response to a set of test items, a model can be developed for the probability of a correct response based on the mastery of the process skills required to successfully respond to the items. This is the basic principle behind cognitive diagnostic modeling (Rupp et al., 2010). Mediation research can also contribute to process research because the mediation variable functions as a process in the narrative of how the level of a dependent variable comes about (Hayes, 2017). It may explain why mediation analysis has become so popular. As far as types of data are concerned, eye movement data are an interesting source of information regarding processes (Cho et al., 2018), because it may be assumed that the mind follows the eyes, or the eyes fixate the stimuli the viewer is processing. Furthermore, brain activation and EEG data can be useful, as well as actions such as clicking and moving on the computer screen to find an answer to a question.

Here we will focus on response times, the time a respondent takes to respond to individual items in a cognitive test. Making use of response times in modeling test data can lead to the identification and measurement of processes, but, as will be discussed, the use of response time information does not necessarily imply it leads to inferences regarding the processes which are involved. The scope of this article comprises modeling approaches in which response times are used and cognitive process inferences can be made. For more general reviews of the use and importance of response time and of time available to make a test, see reviews by Lee and Chen (2011); Kyllonen and Zu (2016) and Schnipke and Scrams (2002).

Response time modeling approaches can be classified into four very broad possibly overlapping and not necessarily homogeneous categories. The categories are partly inspired by an overview made by van der Linden (2009). Before listing the categories, we introduce a symbolic notation for the models:

*T*_*pi*_ for the response time of person *p* and item *i*;
*A*_*pi*_ for the response accuracy of person *p* and item *i*;
← to indicate which variable is the dependent or independent variable; for example, *T*_*pi*_ ← means that response time is the dependent variable.

(a) *Response time models*: response times as the sole end variable (*T*_*pi*_ ←);
(b) *Joint models*: response times as one of the end variables, jointly with another kind of variable (e.g., accuracy) ([*T*_*pi*_, *A*_*pi*_] ←);
(c) *Dependency models*: joint models in which response times and other data (e.g., response accuracy) are jointly modeled with the possibility of dependencies beyond dependencies captured by latent variables and item parameters ([*T*_*pi*_↔*A*_*pi*_]) ←;
(d) *Response times as covariate models*: response times as an origin variable and another kind of variable (e.g., accuracy) as the end variable (*A*_*pi*_←*T*_*pi*_).

An *end variable* is an outcome variable, also called dependent variable, the last variable in a dependency network. For example, in a simple measurement model for speed, the observed response times are modeled as a function of a latent speed variable and item time parameters. More than one variable can have the status of an end variable. For example, response time and response accuracy (correct vs. incorrect) can be joint end variables. An *origin variable* is a covariate, also called independent variable, a variable in the dependency network that is not explained by any other variable. More than one variable can have the status of origin variable.

## Response Time Models

Three subtypes of modeling will be discussed for the *T*_*pi*_ ← case, and thus with response time as the sole end variable: (1) distribution models, (2) explanatory models, and (3) models with response accuracy as a covariate.

### Distribution Models for Response Times

Not only the mean but also the distribution of response times is informative (e.g., Van Zandt, 2002). In most studies response times turn out to be distributed with a variance that increases with the mean. Many types of distributions have this feature or can accommodate this feature: gamma, inverse Gaussian, ex-Gaussian, and ex-Wald, lognormal, Weibull, and Gumble, while in fact also the normal distribution has been used even though it does not have the feature. Distributions are in the first place used as a tool to make a model work, which for some of these distributions means deciding on a link function or a transformation (Lo and Andrews, 2015). However, the distributions have also been interpreted in terms of generating processes and these processes may have cognitive interpretations.

-*Gamma distribution*: is generated when the response process consists of a set of sequential processes with an exponential time distribution, suggesting that the underlying processes are sequential. For example, Maris (1993) has used gamma distribution models to model response times for mental rotation items.
-*Inverse Gaussian distribution*: is generated from an information accumulation process with a single stopping criterion. For illustrations of this and other distributions, see Lo and Andrews (2015).
-*Weibull* and *Gumbel distributions*: are generated from parallel processes with a stopping rule based on the first process that reaches the information accumulation criterion (a decision threshold). The Weibull distribution has been used by Loeys et al. (2011) for a joint model of response time and accuracy.
-*Ex-Gaussian distribution*: is generated by the sum of a normally distributed random variable and an exponentially distributed random variable. It has three parameters: μ and σ for the normal distribution, and τ for the exponential distribution. The exponential distribution explains the skew. The Gaussian component has been interpreted as reflecting automatic processes and the exponential component as reflecting more controlled processes. There also seems to be a relationship of τ with cognitive efficiency (based on the drift rate parameter of the drift diffusion model, see Ratcliff, 1978; Ratcliff and McKoon, 2008) and working memory (Schmiedek et al., 2007). Based on simulation studies by Matzke and Wagenmakers (2009) it seems that all three ex-Gaussian parameters are sensitive to the decision threshold (the boundary separation from the diffusion model) but that primarily τ is sensitive to differences in cognitive efficiency (the drift rate parameter of the diffusion model).
-*Shifted Wald distribution*: is generated by an accumulation process with a certain rate and threshold, and with a shift parameter. The shift parameter can also be added to other distributions to account for the fact that the lower response time boundary is not zero but slightly higher (a zero response time is impossible). The shifted Wald distribution has been used by Anders et al. (2016).

It was Luce's (1986) purpose to derive underlying processes from response time distributions, but he came to the conclusion that the relationship between processes and distribution is not as clear as one would like (p. 173–174), and additionally, differentiating between the distributions is not always easy. The relationship between distributions and processes is also discussed by Van Zandt and Ratcliff (1995).

For the practical purpose of measurement and because it often fits the data very well, the *lognormal distribution* has become popular for cognitive test response times (van der Linden, 2006, 2007) without process interpretation claims. In some other applications, practical considerations have led to an approach based on the proportional hazard principle (e.g., Ranger and Kuhn, 2012, 2014; Ranger and Ortner, 2012; Wang and Xu, 2015; Kang, 2017). Burbeck and Luce (1982) explain that the normal, Gumbel, and ex-Gaussian distributions have a monotone non-decreasing hazard function, while the exponential distribution (a special case of the Weibull) has a constant hazard function, and the Weibull distribution can accommodate a decreasing, constant, and increasing function. Finally, a peaked hazard function applies to the lognormal and the inverse Gaussian. The hazard function approach may be more than just practical for fitting the data. The actual shape of the function (increasing, decreasing, constant, curvilinear) may imply suggestions for the kind of process. As an alternative for the proportional hazards model, the response times can also be categorized so that a generalized linear mixed model approach can be used (Molenaar et al., 2018), and a Box-Cox transformation is another option (Klein Entink et al., 2009a).

### Explanatory Response Time Models

There is a tradition in cognitive psychology to decompose response times based on hypothesized sequential processes (Donders, 1869; Sternberg, 1969). The most extensive work is conducted by Sternberg (1977b, 1985). He started his work with analogy items (Sternberg, 1977a,b) and later extended it to other cognitive problems, such as deductive reasoning problems (Sternberg, 1980, 1986).

His theory, models, and analyses are briefly described here. Suppose an analogy problem “Son is to aunt as daughter is to ?..” (A:B :: C:? ..), with D as the correct response. The hypothesized processes are: encoding, inference, mapping, and application. First, there are three terms to be encoded (“son,” “aunt,” and “daughter”). Second, an inference needs to be made, based on a comparison of A and B (“son” and “aunt”) which implies two differences (sex and generation). Third, mapping consists of comparing A and C (“son” and “daughter”), which implies one difference (sex). Finally, application consists of applying the A:B relationship to C to find D, which implies two differences (sex and generation). A basic assumption in the model is that a difference between terms takes time. To differentiate the number of feature differences to be processed for inference and application and to vary the number of terms to be encoded, one can present the respondents with A and B before the response time is recorded, so that the task requires only the encoding of one term (C), and the feature differences relevant for mapping and application (assuming A and B have already been encoded and an inference is made). The example item with a full item format leads to the following equation:

(1) $$\begin{array}{r} RT=intercept+\;aX_{a}+bX_{b}+cX_{c}+dX_{d}+\varepsilon , \end{array}$$

where *RT* is the response time, *X*_*a*_ = 3 (encoding of A, B, C), *X*_*b*_ = 2 (differences between A and B), *X*_*c*_ = 1 (differences between A and C), *X*_*d*_ = 2 (differences between C and D), and *a*, *b*, *c*, and *d* are parameters referring to the time spent per process, while ε is a residual term. For the reduced item format, with A and B presented before the response time is registered, the equation would be:

(2) $$\begin{array}{r} RT=intercept+\;aX_{a}+cX_{c}+dX_{d}+\varepsilon , \end{array}$$

where *X*_*a*_ = 1, *X*_*c*_ = 1, *X*_*d*_ = 2.

When a person is presented with a large set of problems with different values for the different *X*-variables, regression analyses can be conducted, one per respondent, which is what Sternberg (1977a) did at a time when mixed models were not yet common practice. Based on this approach, he was able to estimate the time each hypothesized process takes per person.

Around the same time as Robert Sternberg did his research, Susan Embretson (Whitely, 1976, 1977) was doing very similar work but with binary accuracy as the dependent variable, using item response (IRT) models. In fact, Fischer (1973) had formulated an IRT model with the potential to do just that. His Q-matrix contains the X-variables from the above equations. Within IRT this has further led to the test design idea (Embretson, 1985), cognitive diagnosis modeling (CDM) (Rupp et al., 2010) and explanatory item response models (De Boeck and Wilson, 2004). An important difference between CDM and the other approaches is that process inferences are discrete (often binary) and refer to mastery of skills that may be related to hypothesized processes; but see Zhan et al. (2018c) for mastery in probabilistic terms. However, because response times are not involved in these approaches, we will not follow up on these developments here.

Explanatory response time models have also been embedded in models discussed elsewhere in this article. For example, Maris (1993) has used item covariates in his gamma model, Klein Entink et al. (2009b) have used item covariates in the hierarchical model of van der Linden (2007) to be discussed in Section Distribution Models for Response Times, and van Breukelen (2005) did the same in a related model. However, such applications with the possibility for process inferences are rather rare, whereas they have clear potential for the study of response times, just as they have for response accuracy. Possibly, the extension of CDM with response time data (Zhan et al., 2017) can lead to a further interest in this approach.

### Response Time as a Function of Response Accuracy

Usually response time is considered as the independent variable for response accuracy and not the other way around. However, there is some literature on how the type of incorrect response is an indication for response time and for the underlying processes. For example, Novikov et al. (2017) hypothesize based on the literature that errors either stem from lack of cognitive control (deemed to be premature responses) and would lead to short response times (error speeding) or from attentional lapses and uncertainty. The study by Novikov et al. (2017) concerns an auditory discrimination task and the use of EEG to locate oscillations in different regions of interest in the brain. On average the response times were shorter for correct responses than for incorrect responses, a common finding for complex attentional tasks (Wilding, 1971; Luce, 1986) and slow errors are found to be an indication of attentional lapses and uncertainty. The empirical results turned out to be roughly in line with the hypothesis about fast and slow errors based on EEG oscillations in regions of interest in the brain known to be informative about the hypothesized processes.

## Joint Models

It has become common practice to register response times for all item responses, so that *parallel data* are available: response accuracy and response time per pair of respondent and item. This allows then for ([*T*_*pi*_, *A*_*pi*_] ←) models, where time and accuracy are joint end variables. The parallel data concept is broader than response time and response accuracy. Although the applications are rare or even non-existing, parallel data can also include eye-movement data, brain activation data (BOLD signals) and EEG data for one or more regions of interest (ROI).

Molenaar et al. (2015) have discussed a broad framework for joint models, called the bivariate *generalized linear item response theory modeling* (B-GLIRT) framework. As shown by Molenaar et al. (2015), these models are basically IRT versions of two-dimensional confirmatory factor analysis (CFA) models: one factor for ability and another (correlated) factor for speed. Guessing and random item parameters are thus far not used in factor models, but they can be and have been included in the IRT versions. The prototypical model in the category is the hierarchical model (van der Linden, 2007), which has inspired related models with a different response time distribution (e.g., Loeys et al., 2011; Wang et al., 2013; Kang, 2017), with a multidimensional extension of the measurement model (Zhan et al., 2018a), and with item response time varying in a systematic way during the test (Fox and Marianti, 2016). An interesting feature of the B-GLIRT framework is that Thissen's (1983) joint model can also be accommodated into B-GLIRT although it may not look like a typical CFA model. Another feature is that polytomous responses can also be dealt with.

The B-GLIRT models are measurement models but not process models. The primary function of response times is to strengthen ability measurement. However, two other types of joint models exist with the ambition to model cognitive processes based on parallel data regarding response time and response accuracy: diffusion models (Ratcliff, 1978) and race models (Townsend and Ashby, 1978). Tuerlinckx and De Boeck (2005) have shown that both these cognitive models can be approximately re-parameterized as item response models and thus as measurement models for test data. Since then, van der Maas et al. (2011) have developed a version of the diffusion model for cognitive test data (see Ranger and Kuhn, 2018, for estimation methods), and Rouder et al. (2015) and Ranger et al. (2014), have developed race models for joint response accuracy and response time data from cognitive tests. The diffusion model and the race model as process models are discussed after the hierarchical model is presented. Finally, there is a beginning research line of using parallel data for cognitive diagnostic modeling (Zhan et al., 2017, 2018b) with the possibility of accommodating local dependencies (Zhan et al., 2018b). These models offer the possibility of extending the hierarchical model and dependency models to another popular type of psychometric models.

### The Hierarchical Model

The most popular method to analyze parallel data is van der Linden's (2007) hierarchical model and it is a member of the B-GLIRT family. Roughly speaking it is a two-dimensional model, with one dimension for accuracy (correct vs. incorrect) interpreted as ability and another dimension for response time (log of response time) interpreted as speed. The model is more complex, because the ability dimension is based on the three-parameter logistic (3PL) model with random items parameters for accuracy as well as for response time. The model is a hierarchical model because of the multivariate distribution for ability and speed and for the item parameters of response accuracy and response time. Furthermore, van der Linden (2009) notes that the ability would be higher and the speed lower if the respondent would make the same test with more focus on accuracy. Therefore, the ability and speed as measured are “effective” ability and speed for an unknown speed-accuracy tradeoff from the part of the respondent. Although the model is very useful as a measurement model, it is not a process model. It is a measurement model with the advantage that the measurement of ability can benefit from the response time information. If the two dimensions are related, the measurement of each of them gains strength from the data for the other.

The assumption of van der Linden (2007) model is that response times follow a lognormal distribution. Loeys et al. (2011) have used the lognormal distribution and the shifted Weibull, while for example Wang et al. (2013) and Kang (2017) have used a semi-parametric proportional hazards model which gives the opportunity to accommodate most types of distributions and deviations from these. As far as the distribution can be interpreted in process terms, the proportional hazard approach can function as an explorative approach for cognitive processes.

### Diffusion Model

The drift diffusion model has been presented in an explicit way as an alternative for the hierarchical model by van der Maas et al. (2011). The model is a modification of the original drift diffusion model (Ratcliff, 1978; Ratcliff and McKoon, 2008; Ratcliff et al., 2016) so that it can be used for multiple-choice data from cognitive tests. The primary process is information accumulation in response to a stimulus (an item) that comes with a binary choice question (e.g., “is the number of asterisks you see smaller or larger than 50?”). The restriction to binary choices is removed in the van der Maas et al. (2011) version. The information accumulation process is not a straight-line process, instead it is a random walk process between two boundaries (one for each response option) with a trend in the direction of one of both but with the possibility to end up at the boundary opposite to the trend because of the random character of the process. When a decision boundary is reached, the corresponding response follows. The trend parameter is called the drift parameter. The other parameters are boundary separation, bias, and non-decision time. The boundary separation represents the speed-accuracy balance (how certain one wants to be before responding), bias depends on where the process starts (in the middle or closer toward and thus in favor of one of the boundaries), and the non-decision time is the time not taken by the information accumulation.

Although the diffusion model is a process model, it is basically a one-process model, with the one process being information accumulation, governed by three parameters (drift, boundary separation, and starting point). The non-decision time is a rest category for processes involved in the perception of the stimulus and the act of responding.

For rather simple binary choice tasks with on average extremely fast responses—much faster than cognitive test responses—it makes sense that only one process is involved, while this is less likely for more complex cognitive tasks as presented in cognitive tests. Information accumulation may be a basic elementary component, but if it is, it would need to be repeated in each of the processes involved in more complex tasks, for example, in each of the processes Sternberg (1977a) has found to play a role in analogy tasks. Such an extension is a serious complication and cannot yet be dealt with in model formulation and estimation.

Still, van der Maas et al. (2011) have shown that latent variable modeling (including item parameters) is possible for the diffusion model assuming just one diffusion process. The major two latent variables in the model are cognitive efficiency (drift rate of the process) which is always positive in the van der Maas et al. model, and cautiousness (boundary separation for the process). Cognitive efficiency makes one respond faster and with a higher probability of a correct response, whereas cautiousness makes one respond slower and with a higher probability of a correct response. Therefore, and roughly speaking one can expect that these two dimensions are a rotation of the ability and speed dimensions of the hierarchical model, with cognitive efficiency in between ability and speed and with cautiousness in between ability and the opposite of speed.

In sum, although the diffusion model has several advantages (a process model, more fine-grained, taking the speed-accuracy balance into account), it is based on a one-process assumption, and as far as the latent variables are concerned, it is roughly speaking a rotation of the hierarchical model. Conceptually speaking, the cognitive efficiency as measured in the diffusion model, shows clear similarities to Spearman's (1927) view on intelligence and how the speed-accuracy balance plays a role in the response process (p. 250).

### Race Models

Race models are based on the notion of a competitive race between accumulators, one for each response option. The Rouder et al. (2015) model has a shift parameter for response time but it has only one latent variable: the ratio of the rate of information gain and response boundary, and for the application Rouder et al. (2015) describe, this one latent variable is highly correlated with effective ability from the hierarchical model. The Ranger et al. (2014) model has two latent variables (but not a shift parameter): one for information accumulation in support of the correct response, and one for misinformation accumulation (supporting the incorrect response). The amount of processing capacity is the sum of these two and accounts for response time, whereas the discrepancy between the two accounts for response accuracy. The authors show that the speed-accuracy trade-off is a complicated function of these two. Because the two latent variables can be approximately re-parameterized as effective speed and effective ability, this race model is equivalent to the recognition of speed and ability as basic latent variables. We have empirical evidence for this conceptual analysis. From our own analysis of data, it was found that for the Ranger et al. latent variables the multiple correlations with effective ability are 0.886 and 0.833 (two different sets of items were used) and with effective speed they are 0.979 and 0.962. In other words, although the models have very different functional forms, the latent variables that are being extracted belong roughly to the same two-dimensional space.

The race models share with the diffusion model that they are process models, that they are more fine-grained, and that they have a solution for the speed-accuracy issue, but as far as latent variables are concerned, they seem to work with roughly the same two-dimensional space as the hierarchical model. In other words, the difference with the hierarchical model is primarily an interpretation difference. The diffusion model and race models both assume one primary process: either information accumulation between boundaries, or a race among different accumulators.

## Local Dependency Models

Local dependency models are models in which response time and response accuracy are jointly modeled but in which they are also related to each other beyond the relationship of their corresponding latent variables and item parameters so that they imply or can explain an extra dependency (of the type [*T*_*pi*_ ↔ *A*_*pi*_] ←). While *T*_*pi*_ and *A*_*pi*_ are end point variables, they also are covariates to explain the local dependency.

### Types of Models

There is clear evidence for local dependencies between response time and accuracy (Bolsinova and Maris, 2016). The inclusion of dependencies in a joint model can be realized through the introduction of local dependency parameters or through models with different classes of responses (based on different response mechanisms). The former models are *latent variable models with remaining dependencies*. Either the item response time has a direct effect on the corresponding item accuracy (Bolsinova et al., 2017a; De Boeck et al., 2017) or vice versa (van der Linden and Glas, 2010), or the relationship is modeled as a symmetrical residual dependency. The alternative type of models are class models with two *classes of responses* corresponding to two response modes: a fast mode and a slow mode. The classes are classes of item responses (not of items and neither of persons), each with a different model and thus with different processes to arrive at a response. Examples of such models are described by Partchev and De Boeck (2012) (for manifest classes) and by Molenaar and De Boeck (2018), Wang and Xu (2015), Molenaar et al. (2016) for latent classes.

In the models presented in the former two articles with class models, either the observed item response time determines which model applies for accuracy (Partchev and De Boeck, 2012) (it is a manifest class model) or the item response time is a covariate for the probability of the model that applies for accuracy (Molenaar and De Boeck, 2018) (it is a latent class model). In both these models there is only one sub-model (one class) for response times, but there are two for accuracy. Which of the two applies depends on the response time, in a deterministic way in the former model and in a stochastic way in the latter.

In the other two models the response classes are associated with different models for response accuracy and response time. In the Wang and Xu (2015) model, one class represents the regular problem solving process and the other class is a rapid guessing class, while in the Molenaar et al. (2016) model, the two classes represent fast and slow problem solving processes (with a Markov transition between the two), respectively, but none of the two corresponds to guessing.

Two other models may seem similar to the latter two, but they are in fact person class models and not response class models. First, Meyer (2010) has also published a model for response time and response accuracy with two classes, a regular problem solving class and a rapid guessing class, for problem solvers and rapid guessers. Second, Jeon and De Boeck (2018) also work with person classes, each with its own accuracy model and with item response times as covariates of the class probabilities. The resulting classes are interpreted by the authors as a regular problem solving class and one or two automatic knowledge retrieval classes.

### Findings

Based on the latent variable models with remaining dependencies, the main finding is a negative dependency between response time and response accuracy. Fast responses (short response times) have a higher accuracy (Bolsinova et al., 2017a,b; De Boeck et al., 2017). The dependency cannot be explained by the fact that easy items require less response time because the relationship across items (and persons) is taken care of through the item parameters (and the latent variables). The results are supported by the response class models with a fast and slow class. Items are easier in the fast response class than in the slow item response class (Partchev and De Boeck, 2012; DiTrapani et al., 2016; Molenaar et al., 2016; Molenaar and De Boeck, 2018). The rapid guessing mixture model cannot explain these results because it implies a positive dependency (slower responses are more correct). It is possible that the two types of response class models inform us about different underlying phenomena in the same data. Rapid guessing is considered an important phenomenon in educational measurement. It has been linked to lack of motivation, and in line with this hypothesis a response time effort (RTE) index has been developed (Wise and Kong, 2005; Wise and Gao, 2017) to identify motivation issues.

The negative dependency does not show in all studies, for example, in one of the two datasets in Bolsinova et al. (2017b), the dependency is positive. The exceptions can be explained by another rather robust finding that the dependency is positively correlated with the difficulty of the items (Meng et al., 2015; Bolsinova et al., 2017a,b; De Boeck et al., 2017; Molenaar and De Boeck, 2018). The easier (more difficult) the items are the stronger (weaker) the negative dependency is, and for more difficult items the dependency can be positive.

The negative dependency can be interpreted as the consequence of attention variation during the test. This would imply a variation of cognitive efficiency and thus a higher (lower) accuracy paralleled by shorter (longer) response time. The link with item difficulty can be explained if one assumes, in line with the diffusion model, that dominant responses are faster. The easier an item is, the more dominant the correct response is, and thus faster. For the difficult items, there may be one or more dominant incorrect responses raising the chances of an incorrect response being faster. Therefore, a variation of cognitive efficiency may lead to an association of fast with correct or with incorrect, depending on the difficulty of an item.

There are some alternative explanations for the same findings. First, on average easy items come with faster responses, but if easiness also depends on the respondent this would lead to a negative dependency between response time and response accuracy. At the same time, difficult items come with slower responses, but it is likely that respondents guess more on difficult items, which would lead to fast responses with a small probability of being correct. Second, it is also possible that, again on average, for easy items one relies more on automated processes, such as knowledge retrieval, which can be very fast, whereas difficult items require more controlled processing, which takes time. The latter explanation can be found in Goldhammer et al. (2014) for results that will be discussed in the next section on studies with response time as a covariate. For a further discussion of possible explanations, see Bolsinova et al. (2017c).

Based on the studies cited here, the residual dependencies are a robust finding, in low-stakes and high-stakes tests, for open-ended as well as multiple-choice items, for children and adults, for educational tests as well as for intelligence tests. They are an intriguing phenomenon in the investigation of cognitive processes because they are derived from a more fine-grained analysis than the common models with latent variables and item parameters. Latent variables inform us about rather general individual differences in speed and ability and their association seems to vary depending on the test (Schnipke and Scrams, 2002; Klein Entink et al., 2009c; van der Linden, 2009). They can stem from differences in the speed-accuracy balance and other confounding variables. With respect to correlations across items, overall item differences in time intensity and difficulty and the fact that more difficult items take more time are rather self-evident findings. However, the dependencies are a new category of findings obtained after controlling for general differences and associations across persons and items; they refer to the more specific relationship between response time and accuracy (Bolsinova et al., 2017c).

One further and even more specific finding, although not based on joint modeling of response times and response accuracy, but on double-centering of response times instead (an explorative technique) is that the residual relationships between response time and difficulty may be curvilinear (Chen et al., 2018). The curvilinear relationship including its precise shape is confirmed with a fine-grained modeling approach by Bolsinova and Molenaar (2018). Naumann and Goldhammer (2017) also obtained curvilinear relationships with a method described in Section Local Dependency Models, and van Breukelen (2005) found indications of curvilinearity for some types of items with a related model.

Another and very recent joint latent variable model with dependencies is the generalized speed-accuracy response model for dichotomous items (van Rijn and Ali, 2017, 2018). It is a model with only one latent variable (a capacity variable) for when a scoring rule is used described by Maris and van der Maas (2012). Starting from the scoring rule, a corresponding model is formulated, by way of reversed engineering. The scoring rule implies that correct (incorrect) responses are rewarded (penalized) more the shorter the response time is. Responses, whether correct or incorrect do contribute less to the score the slower they are. When all the available time to respond is used (response time equal to the time limit) the response has no effect on the score. The model is at the same time a model with local dependence between response time and response accuracy, which is not surprising given that it is a model for a scoring rule that combines correctness and response time. Interestingly this model is applied by the authors to data from respondents who were not aware of the scoring rule. Therefore, the implicit assumption is that the rule they were using reflects their actual speed-accuracy balance. The speed-accuracy balance is of a different kind than the one defined by the boundary separation in the diffusion model. The latter implies that the larger the boundary separation is, the larger the value discrepancy is between a success and a failure. Instead, following the Maris and van der Maas scoring rule, the value of success and failure depends on the response time. The model does not allow for individual differences and item differences with respect to the speed-accuracy balance, but such an extension could lead to an estimation of the balance. A further interesting implication of the model is that the relationship between response probability and response time is curvilinear.

The findings from the class models are partly overlapping with those from latent variable models with residual dependencies in that the negative dependency and the link with item difficulty are supported as explained earlier. On the other hand, the class models seem to provide evidence for a dual-processing view. This is easy to understand for rapid guessing as a processing mode (Meyer, 2010; Wang and Xu, 2015), even though it might be necessary to distinguish between rapid guessing and cheating (Wang et al., 2018) because cheating can also be fast. Class models may be more difficult to understand for other distinctions between processes (if not prior suspects such as rapid guessing or cheating are available). A first obstacle is that the latent variable for accuracy is the same or highly correlated in the two classes in class models for slow and fast responses (Partchev and De Boeck, 2012; Coomans et al., 2016; DiTrapani et al., 2016; De Boeck et al., 2017; Molenaar and De Boeck, 2018). It means that, although the processes seem different, as one may infer from a difference in item parameters, the underlying abilities cannot be differentiated. When a respondent switches from one mode to another, which is modeled through a Markov model in Molenaar et al. (2016), an empirically not distinguishable ability is being used. This may seem odd, but it is possible indeed that, for example, the abilities for automated processing and controlled processing are empirically extremely highly correlated and nearly identical, even though the actual processes are different. A second obstacle is that the differences between the two classes have not much been explored in terms of item features or kinds of error. Based on the only effort we know of (Coomans et al., 2016), there is evidence for a qualitative difference between the response errors in the fast and slow response classes. For the two example items (multiplication items) given in Table 5 of the article, fast errors seem to be typos or negligent responses based on the correct or a related arithmetic operation, whereas slow errors can be reconstructed based on an unrelated kind of operation. For example, for 100 × 3000 = ?, 3,0000 is a popular fast error, and 400,000 and 1,300,000 are more typically slow errors. Similarly, for 2 × 80?, 40 is more popular as a fast than as a slow error and the reverse is true for 600. Whereas, fast errors seem to be slips, slow errors seem based on complicated incorrect operations or slow guesses.

## Response Times as Covariate Models

Finally, there are studies in which response times are used as a covariate, in all cases with response accuracy as the dependent variable (models of the type *A*_*pi*_ ← *T*_*pi*_). Response time is the origin variable and accuracy is the end variable. We will first discuss models inspired by the speed-accuracy tradeoff (SAT) and next the generalized linear mixed model (GLMM) approach of Goldhammer and colleagues will be covered. A combination of both can be found in van Breukelen (2005) and his analysis of mental rotation data.

### SAT-Based Models

Perhaps the most well-known phenomenon that relates response time to accuracy is the speed-accuracy trade-off (Heitz, 2014). The SAT implies that the success rate shows an exponential growth to a limit as a function of time. The curve has been described by Wickelgren (1977) and is very similar to the curve that can be derived from the diffusion model (Wagenmakers et al., 2004). Lohman (1989) has used the curve for test data and has estimated the corresponding person parameters, such as the growth rate and the upper asymptote. It does make sense that with increasing time available, the accuracy rate goes up. A quite different question is whether the success rate goes up with the time a respondent takes to respond.

Roskam (1987) and Verhelst et al. (1997) make the assumption that a similar growth curve as the SAT curve applies to the time a respondent takes to respond (Roskam, 1987, 1997) and to minus the actual speed of a respondent (Verhelst et al., 1997). Wang and Hanson (2005) make the same assumption as Roskam although for a more complex model. A very nice feature of the Wang and Hanson (2005) model and of Lohman's (1989) approach is that the growth rate can be interpreted as speed (accuracy gain per unit of time, analogous to miles per hour) and the upper asymptote can be interpreted as power in the sense of the maximum accuracy one can reach. While it is undoubtedly true that the probability of success increases as a function of releasing time pressure or extending the available response time (e.g., Semmes et al., 2011; Davison et al., 2012; Goldhammer and Kroehne, 2014; Goldhammer et al., 2017; Chen et al., 2018), it also seems empirically the case that the accuracy curve does often not increase with the observed response time, as will be discussed in the following.

### GLMM Based Covariate Models

In a series of studies, Goldhammer and colleagues (Goldhammer et al., 2014, 2015, 2017; Naumann and Goldhammer, 2017) have investigated the relationship of time on task with response accuracy, inspired by a dual-processing theory. The basic findings obtained with GLMM are that the association between response time and response accuracy controlling for the latent accuracy variable and for accuracy item parameters depends on the kind of task. However, it was always the case that the association is less negative (or more positive) for more difficult items. This was true for reading and problem solving tasks (Goldhammer et al., 2014), Raven items (Goldhammer et al., 2015), lexical decision tasks (Goldhammer et al., 2017), and digital reading (Naumann and Goldhammer, 2017). These results are perfectly in line with the results obtained from local dependency models, and they are also in line with findings by Jeon and De Boeck (2018) that faster than expected response times have a positive covariate effect on the probability of belonging to respondent classes where easy items are even easier, which are interpreted as knowledge retrieval (vs. problem solving) classes in line with the dual-processing hypothesis. The difficulty related dependencies are interpreted from the hypothesis that easy tasks are more amenable to automatization. Because in the studies by Goldhammer and colleagues the relationship between response time and response accuracy was more negative for respondents with high values on the accuracy latent variable, higher levels of skill are also assumed to correspond with higher levels of automatization.

### Discussion and Conclusion

We will first discuss the general finding of local dependency, followed by some considerations regarding cognitive process modeling based on response times. For each of the points, conclusions and suggestions for further directions will also be formulated.

The general finding of local dependency between response time and response accuracy is important for at least three reasons. First, the dependency is a violation of measurement invariance because the dependency implies that ability and speed cannot be measured independently. It is important to investigate how large the resulting distortions are. It is possible that the established violations do not cause large measurement distortions. Second, although the local dependency does not give a direct process indication, it can be interpreted as an indirect indication of the main type of processing: automated vs. controlled processing. The distinction, and thus the dual-processing theory, must not necessarily be interpreted as a dichotomy, it can also be interpreted as a continuum. When interpreted as a dichotomy, it corresponds to the class models for response time and response accuracy. When interpreted as a continuum, it corresponds to latent variable models with residual dependencies and to the research line of Goldhammer et al. Third, the dependency seems to have a specific shape indicating that up to a certain point longer response times are associated with an increasing accuracy, after which longer response times become associated with a decreasing accuracy. To be clear, this is not a result based on the relationship between the latent variables; instead it is based on the local dependencies after controlling for latent variables. Following the results from Chen et al. (2018) the turning point comes earlier if the test is more knowledge based and less reasoning based. The shape of the curve may reflect the cost of time and effort on the speed-accuracy tradeoff. Early on in the response process the cost of spending more time is compensated by an increasing chance to find the correct response, but the longer it takes to find the correct response the higher the cost becomes while the perceived chance of finding the correct response may decrease so that the expectation of a correct response does no longer compensate for the cost of effort. This may not play a role for simple cognitive tasks with fast responses, but it seems more likely for problems as presented in a cognitive test, especially when the test has a global time limit. Future research should take the increasing cost of time and effort into account.

Most of the cognitive test research related to response times is focused on measurement and improvement of the quality of measurement, either making use of response times as collateral information for the ability to be measured or to identify and solve issues. One of the major issues is the speed-accuracy trade-off. Working at a slower or faster rate can reflect a natural pace but it may also be induced by a chosen speed-accuracy balance with consequences for the accuracy of responses and thus for ability estimation, and a faster or slower rate can also have consequences for speededness toward the end of the test. Unless an experimental design is used with a manipulation of the available time, it is not possible to investigate and measure the effects of the SAT. However, experimental manipulations do not inform us about the speed-accuracy balance a respondent chooses when taking a test. The diffusion model seems to give an answer to that important question. It may be a valid answer for the simple two-choice tasks, but it is unclear whether it does for cognitive tests. Further, the assumption of the diffusion model is very similar to Spearman's (1927) assumption that speed and accuracy are governed by cognitive capacity and trading accuracy against speed. Consequently, there is no room for speed as a capacity or as a natural pace variable. Instead there is just one cognitive capacity which determines fast and accurate responses, except for a possibly interfering attitude: the speed-accuracy balance the respondent chooses to work with. To summarize, one cannot simply transpose the diffusion model to cognitive test data and make inferences about the SAT based on that model. Future diffusion model based research should take the nature of cognitive tests into account.

Another major issue is rapid guessing, due to lack of motivation, or due to strategic considerations such as gaining time in order to focus on items with a better perceived chance of success. Rapid guessing is an important practical measurement problem, but it does not inform us about the cognitive processes that play a role when the respondent does work on finding a correct response. It is surprising that response time decomposition models are not used more for cognitive tests, in the line of the cognitive process research by Robert Sternberg. Instead, this more differentiated research is represented in cognitive diagnostic modeling and thus in research and measurement based on response accuracy instead of response time (but see Zhan et al., 2017), whereas response times have a natural relevance for process research. It would be of interest for future research to focus more on response time decomposition models for cognitive test data, beyond the issue of rapid guessing. A combination of response time modeling with cognitive diagnostic model is an alternative and promising avenue for research.

In the future, process research can also come from other types of parallel information, such as eye movement data, recording of actions during the responding process (through clicks and moves on the computer screen), and brain imaging and EEG data. One of the important ongoing trends is the use of data analytics to unravel processes based on recorded actions during the time between the item presentation and the actual response. It is too early for a bet on which approaches will lead to breakthroughs. We should also consider that processes can be so complex and highly variable that it may not pay off to identify what the specific processes are and how they relate, and that it may be more efficient to assess cognitive processes on a higher level of abstraction, for example, how much they are based on automated vs. controlled processes. To summarize, the inclusion of other types of data beyond response times, such as eye tracking data and brain imaging may lead to important novel findings, but, perhaps choices have also to be made regarding the detailed or more general nature of processes one wants to investigate. A good compromise between specificity and generality of processes seems desirable.

## Author Contributions

All authors listed have made a substantial, direct and intellectual contribution to the work, and approved it for publication.

### Conflict of Interest Statement

The authors declare that the research was conducted in the absence of any commercial or financial relationships that could be construed as a potential conflict of interest.
